# “Old wine in a new bottle” - post COVID-19 infection, central serous chorioretinopathy and the steroids

**DOI:** 10.1186/s12348-021-00244-4

**Published:** 2021-05-14

**Authors:** Srinivasan Sanjay, Poornachandra B. Gowda, Bhimasena Rao, Deepashri Mutalik, Padmamalini Mahendradas, Ankush Kawali, Rohit Shetty

**Affiliations:** 1 Department of Uvea and Ocular Immunology, 121/C, Chord Road, Narayana Nethralaya, Bangalore, India; 2 Department of Retina, 121/C, Chord Road, Narayana Nethralaya, Bangalore, India; 3 Chest and Maternity Centre, 878, 5th Block, Near Bashyam Circle, Rajaji Nagar Bengaluru, Karnataka 560010 India; 4 Vikram Hospital, Anne’s College, No.71/1, Millers Road, Bangalore, India; 5 Department of Neuro-ophthalmology, Cornea and Refractive Surgery, 121/C, Chord Road, Narayana Nethralaya, Bangalore, India

**Keywords:** Corona virus disease-19 (COVID-19), Ophthalmic manifestations, Central serous chorioretinopathy, Spectral Domain Optical Coherence Tomography (SD-OCT), Inhalational steroids, Oral steroids

## Abstract

**Introduction:**

Corona virus disease (COVID-19) pandemic can cause myriad of ocular manifestations.

We report a case of unilateral multi focal central serous retinopathy, post COVID-19 infection in an Asian Indian female.

**Case presentation:**

A 42-year-old female presented to us with unilateral blurring, in the right eye (OD), 12 days after COVID-19 infection. She had fever, chills, shortness of breath and cough with tiredness and was COVID- RT PCR positive. She was administered intravenous and oral antibiotics with injection heparin/remdesivir, during her 7 day stay at the hospital. She was also on steroid inhalers. She had no systemic history of note.

On ocular evaluation, her corrected distance visual acuity was 20/40 in OD and 20/20 in left eye (OS). Anterior segment was normal. Anterior vitreous was clear. Fundus examination of the OD showed central serous retinopathy (CSCR) with OS being normal.

**Conclusion:**

CSCR can occur post COVID-19 due to steroid administration and physicians administering it should be aware of this and refer the patients to an ophthalmologist earlier.

## Introduction

Severe acute respiratory syndrome virus 2 (SARS-CoV2) infection resulted in a global pandemic of Coronavirus disease 2019 (COVID-19). Wuhan in China was the first place of the outbreak in December 2019. As of **23 March 2021**, there have been **123,419,065 confirmed cases** of COVID-19, including **2,719,163 deaths**, reported to WHO. As of **19 March 2021**, a total of **397,950,709 vaccine doses** have been administered [1]. Conjunctival involvement, cotton wool spots (CWS) and retinal hemorrhages, central retinal artery/vein occlusion, ophthalmic artery occlusion, panuveitis, papillophlebitis, multifocal chorioretinitis and Adie’s syndrome are the ophthalmic manifestations associated with COVID-19 infection [2–6].

We describe an unique case of unilateral multifocal central serous retinopathy (CSCR) in a patient who had just recovered from COVID-19 and had been treated with inhalational and oral steroids.

## Case presentation

The procedures followed were in accordance with the ethical standards of the responsible committee on human experimentation (institutional or regional) and with the Helsinki Declaration of 1975 as revised in 1983. The patient’s written and informed consent was obtained and the study was approved by the hospital ethics committee.

A 42-year-old Asian Indian female presented to us with unilateral blurring, in the right eye (OD), 12 days after COVID-19 infection. Prior to presentation to us, she had fever, chills, shortness of breath and cough with generalized fatigue. Her physician noted that she was afebrile, oxygen saturation was 97% with few crepitations in her lungs and heart rate was 132/min. Complete blood count (CBC) was within normal limits, erythrocyte sedimentation rate (ESR) 35 mm /hr., random blood sugar (RBS)-118 mg/dl, COVID-19 rapid antigen test was negative.

**Fig. 1:**
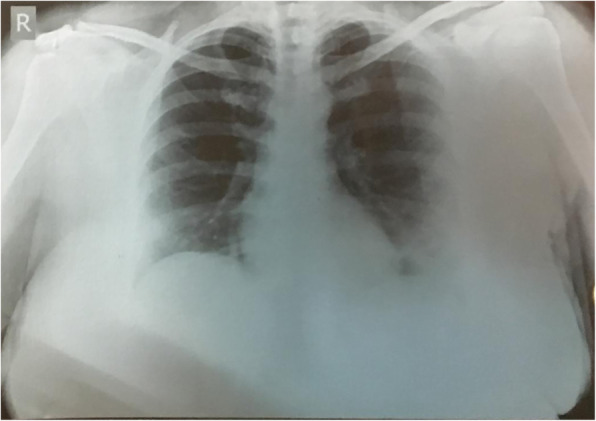
Chest X ray PA view of the lung showing bilateral ground glass opacities with left lung consolidation during her admission

Investigations done at the local hospital a day later showed upper respiratory swab for COVID-19 using real time polymerase chain reaction (RT-PCR) was positive and B-beta (Corona virus) CoV specific target gene and severe acute respiratory syndrome- corona virus 2 (SARS-CoV2) specific target gene were detected, WIDAL test negative, urine analysis was within normal limits. Chest X ray of the lung showed bilateral ground glass opacities (Fig. 1). Based on the records that were available with the patient, we in Table 1 show the medications administered during her stay at a local hospital.

**Table 1:** Shows medications administered to the patient while she was admitted at the local hospital

Route	Drug	Dose/duration
Intravenous	Cefoperazone+ Sulbactam	1000 mg + 1000 mg for 4 days
Subcutaneous	Heparin	5000 units every 8 h for 3 days
Intravenous	Remdesivir	200 mg loading dose, then 100 mg a day for 4 days
Oral	Dexamethasone	6 mg daily for 7 days
Oral	Azithromycin	500 mg for 7 days
Oral	Doxycycline	100 mg twice daily for 7 days
Oral	Montelukast and Levocetrizine combination	(10 mg) and (5 mg) for 7 days
Oral	Vitamin C	1 g for 7 days
Oral	Pantoprazole	40 mg once daily for 7 days
Oral	Ivermectin	12 mg once daily for 7 days
Inhalation	Oxygen	2 litres/minute for 7 days
Inhalation	Formoterol fumarate dehydrate and budesonide 200 combination	(6 mcg) and 9200mcg) twice daily, which was continued even after discharge
Inhalation	Salbutamol rotacaps	four times daily

She had no systemic history of note

At the time of discharge she was switched to oral steroids (methylprednisolone) 16 mg once daily till her presentation to us

Legends: *mg* milligram; *mcg* microgram

On ophthalmic examination, her corrected distance visual acuity was 20/40 in OD and 20/20 in the left eye (OS). The intraocular pressure was 15 and 18 mmHg in OD/OS respectively. Examination of the anterior segment was normal in both eyes (BE). Fundus evaluation, OD showed absent foveal reflex with serous elevation of the retina with ring reflex at the macula. The OS was within normal limits.

**Fig. 2:**
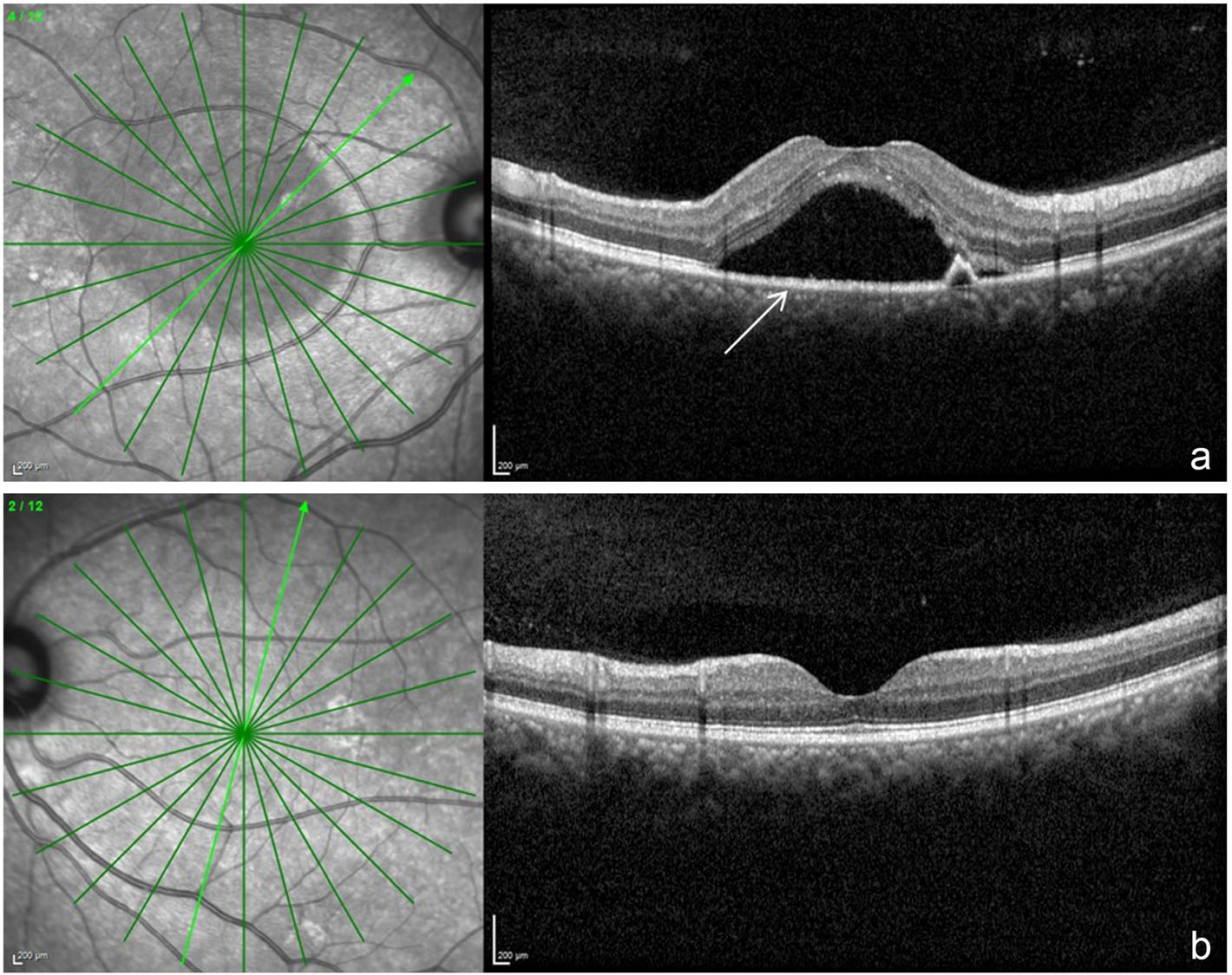
**a** shows a spectral domain optical coherence tomography scan across the macula of the OD. The white arrow points to a doom shaped elevation which is the serous retinopathy. Also in the scan is a smaller doom which represents retinal pigment epithelial detachment. **b** shows the normal scan of OS

**Fig. 3:**
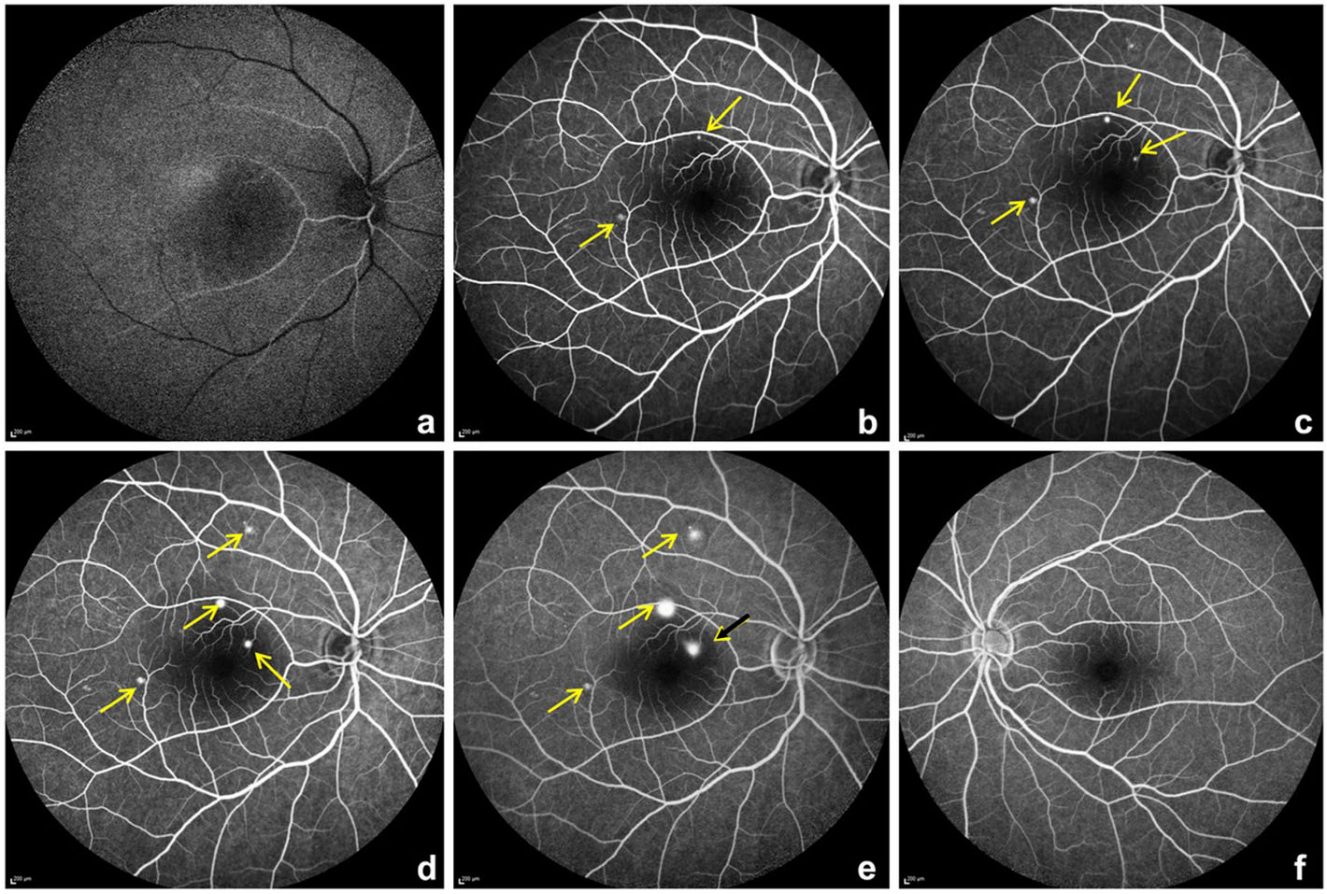
**a**-**e** fundus fluoroscein angiography (FFA) of the OD from early phases a,b to later phases **c**-**e**. The yellow arrows point to a pinpoint leak initially and increasing in size in later phases. The black arrow with yellow arrow head adjacent to optic disc shows a mixed inkblot and smoke stack pattern. f- shows normal left eye

A spectral domain optical coherence tomography (SD-OCT) scan on the Spectralis™ (Heidelberg Engineering, Heidelberg, Germany) of the OD showed hyper-reflective dots in the posterior vitreous, altered foveal contour with serous detachment in the macula and with pigment epithelial detachment (Fig. 2 a). OS was normal (Fig. 2 b). Fundus fluoroscein angiography (Fig. 3a-e) showed an arm-retina time of 18 s with multiple hyperfluoroscent spots seen in the macula which increased in size and intensity in later films in an inkblot pattern characteristic of central serous retinopathy in OD. One of the lesion showed a mixed “smoke stack” and “ink blot” appearance (Fig 3e).

At the time of ocular presentation, investigations showed serum Ferritin 87.90 U/L (females 11–307), Procalcitonin 0.032(< 0.5), mild leucocytosis **11,600** (4500–11,000), ESR-**35** (< 20 mm/hr), CRP **10.7** mg/l(< 3), Immunoglobulin (Ig) G antibodies to nucleocapsid antigen of SARS-CoV2 were positive **4.41** (< 1.0 non reactive).

Based on the clinical and imaging findings, a diagnosis of unilateral OD multifocal central serous chorioretinopathy (CSCR) was made. In consultation with chest physician the inhalational and oral steroids were stopped.

**Fig. 4:**
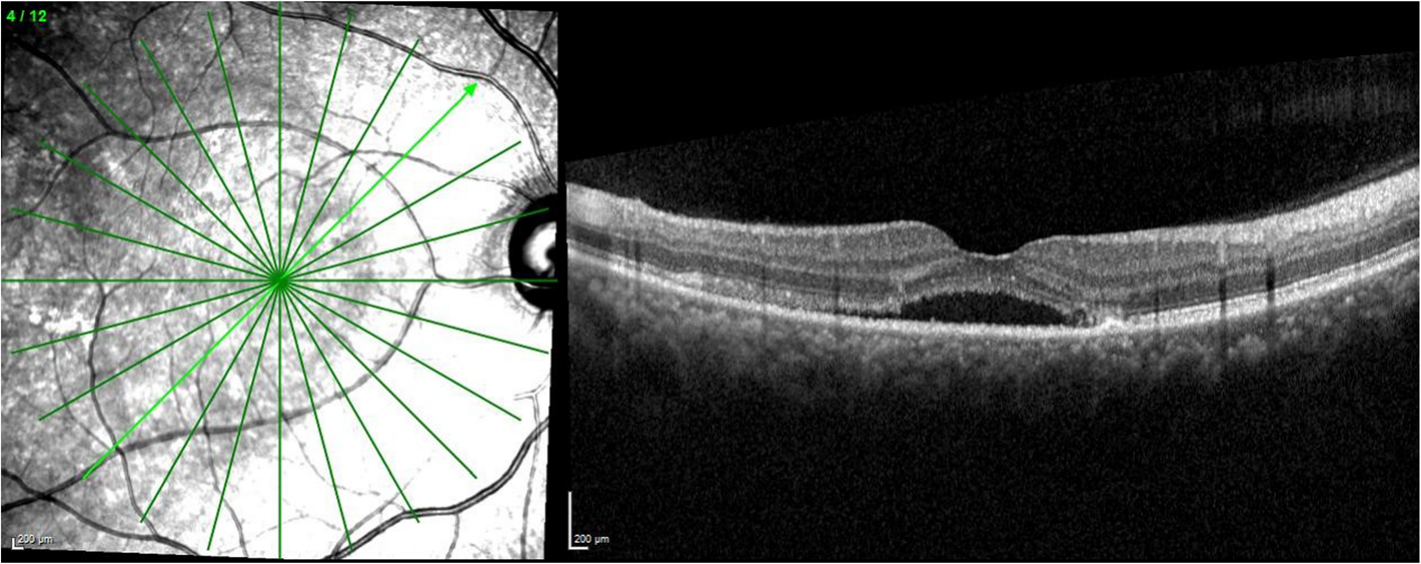
shows a spectral domain optical coherence tomography scan across the macula of the OD with reduction of the sub-retinal fluid and the pigment epithelial detachment a month later

One month later, there was an improvement in her vision to 20/25 in OD, OCT showed reduction of the sub-retinal fluid and the hyper-reflective material and resolution of the pigment epithelial detachment (Fig. 4)

We would like to report for the first time a case of a unilateral multifocal CSCR in an Asian Indian post COVID-19 treatment.

Published data show that SARS-CoV-2 binds to the host cells via the angiotensin converting-enzyme (ACE) 2 receptor [7]. Endothelial cells become vulnerable when the ACE 2 receptors are expressed and binding of SARS-CoV-2 may cause systemic endothelial dysfunction. All major organs like the lungs, heart, veins, and arteries have higher density ACE 2 receptors. Endothelial dysfunction leads to vasoconstriction, ischemia, tissue edema, and a procoagulant state secondary to endothelial alterations including endothelitis [7].

The exact pathophysiology of ocular transmission of the virus remains incompletely understood, although there is preliminary evidence of SARS-CoV-2 being detected in ocular secretions. The ocular tropism of the virus and its potential to cause localized ocular disease are worth considering [8].

Steroids may be necessary to manage the post COVID-19 systemic manifestations including the cytokine storm.

Our patient was administered oral and inhalational steroids during her stay at the local hospital. CSCR occurs or is aggravated by administration of corticosteroids irrespective of the route of administration. Steroids when used topically for skin conditions, intra-articular, intravenous, intramuscular, oral, epidural, intranasal and inhalation are all associated with CSCR [9, 10].

It is postulated that the blood retinal barrier may be damaged, with damage to retinal pigment epithelial pump and hyperpermeability of choriocapillaries leading to CSCR.

CSCR can develop secondary to exogenous corticosteroids several years later.

There may be a temporal correlation between the use of a corticosteroid nasal spray and the development of CSCR. Posterior sub capsular cataract can occur after nasal/inhaled steroids.

Cessation of inhalational steroids can lead to resolution of CSCR, which also happened partially in our patient [11].

The choroid has extensive choriocapillaries whose role is to supply oxygen and nutrients to the outer retina which has no vascular network. Glucocorticoids possibly enhance the fibroblasts proliferation with compromised capillary function leading to their fragility. Corticosteroids may affect choroid, Bruch’s membrane, or the retinal pigment epithelium [12].

Postulates on the mechanism of CSCR include vascular auto-regulation via increased transcription of adrenergic receptors or potentiation of vascular reactivity, effects from steroid-induced systemic hypertension, or a prothrombotic effect. Inhibition of collagen synthesis in Bruch’s membrane may be another mechanism. The barrier function of retinal pigment epithelium (RPE) may be compromised due to impaired ion and water transport. The role of the RPE in CSCR pathogenesis remains poorly understood. Increased tissue hydrostatic pressure in the choroid can cause the barrier function of the RPE to be compromised and lead to areas of fluid accumulation between the retina and the RPE which can also lead to pigment epithelial detachment(s) which also represent a form of RPE decompensation [13]

Some refer to the pinpoint areas of leakage seen in acute CSCR as “micro-rips” or “blowouts”.

CSCR is a self-limiting condition; various modalities of treatment have been described in the literature for the recurrent cases. One month after stopping steroids (inhalation and oral) there was a significant improvement in patient’s visual acuity and SD-OCT findings. Subsequently she was lost to follow up.

## Conclusion

We hereby describe a patient with unilateral serous chorioretinopathy post COVID-19 infection, who developed ophthalmic manifestations 12 days after she was discharged from the hospital, after having tested negative for COVID-19. Patients with COVID-19 should be warned about possible ophthalmic sequelae even after their systemic recovery. Physicians treating COVID-19 should be aware of these important sequelae and refer the patient to an ophthalmologist for timely intervention.

## Data Availability

Available on request

## Abbreviations

SARS-CoV2: Severe Acute Respiratory Syndrome Corona Virus 2
COVID-19: Coronavirus disease 2019
CWS: Cotton wool spots
OD: Right eye
CBC: Complete blood count
ESR: Erythrocyte sedimentation rate
RBS: Random blood sugar
RT-PCR: Real time polymerase chain reaction
OS: Left eye
SD-OCT: Spectral domain optical coherence tomography
CSCR: Central serous chorioretinopathy
Ig: Immunoglobulin
ACE: Angiotensin converting-enzyme
RPE: Retinal pigment epithelium
